# Percutaneous Left Atrial Appendage Closure in a Patient With Acquired Von Willebrand Disease and Atrial Fibrillation

**DOI:** 10.7759/cureus.63936

**Published:** 2024-07-06

**Authors:** Alessandro Giaj Levra, Gulrays Jamie, Ottavia Cozzi

**Affiliations:** 1 Department of Biomedical Sciences, Humanitas University, Milan, ITA; 2 Cardio Center, Humanitas Research Hospital, Scientific Institute for Research, Hospitalization and Healthcare (IRCCS), Milan, ITA

**Keywords:** pre-operative management, prevention of ischemic stroke, left atrial appendage occlusion, new-onset atrial fibrillation, acquired von willebrand disease

## Abstract

Left atrial appendage closure (LAAC) can be used to prevent embolic events in patients with atrial fibrillation who cannot tolerate oral anticoagulants. LAAC has not yet been performed in patients with acquired von Willebrand syndrome. A 74-year-old male with von Willebrand disease presents to the emergency department because of palpitations. Atrial fibrillation with congestive heart failure, hypertension, age ≥75, diabetes, stroke, vascular disease, age between 65-74, and female sex (CHA2DS2-VASC) of 4 was diagnosed. Oral anticoagulation was withheld because of a past medical history of major bleeding events despite treatment of the underlying bleeding diathesis. Therefore, LAAC was considered for stroke prevention. However, the procedure was delayed due to abnormal coagulation cascade levels. Because of the ineffectiveness of treatment and persistently low levels of factor VIII and von Willebrand factor (vWF), the von Willebrand disease hypothesis was abandoned, prompting a new diagnosis for the bleeding disorder. Rapid clearance of factor VIII and vWF, the good response to intravenous immunoglobulins, and the presence of monoclonal gammopathy of undetermined significance allowed the diagnosis of acquired von Willebrand syndrome. After administration of immunoglobulins, factor VIII and vWF levels were normalized, and the LAAC was performed. The patient was discharged on low-dose aspirin. At the nine-month follow-up, the patient did not experience bleeding or embolic events. Stroke prevention in patients with atrial fibrillation and increased bleeding risk requires alternatives to oral anticoagulation. LAAC can be safely performed in patients with acquired von Willebrand syndrome and atrial fibrillation.

## Introduction

Atrial fibrillation (AF) is a potent risk factor for thromboembolic events [1]. Although oral anticoagulation (OAC) for stroke prevention is generally indicated for patients at high thrombotic risk [2], its indication must be carefully weighed up with the patient-specific bleeding risk [3]. Percutaneous left atrial appendage closure (LAAC) is a non-pharmacologic alternative for stroke prevention in patients who cannot tolerate chronic OAC [2,4].

Acquired von Willebrand syndrome (AvWS) is a rare condition associated with high bleeding risk. It usually occurs in patients with underlying lymphoproliferative disorders, most commonly monoclonal gammopathy of undetermined significance (MGUS) [5]. Circulating monoclonal antibodies in MGUS bind to the von Willebrand factor (vWF); the vWF-immunoglobulin complexes accelerate the clearance of vWF by the reticuloendothelial system [6].

In this report, we present the first case of LAAC in a patient with AF and AvWS secondary to MGUS. PubMed was searched for English-language articles published from database inception to May 2024, using the keywords (“acquired von Willebrand syndrome”) AND (“left atrial appendage closure”) but no results were retrieved.

## Case presentation

**Figure 1:**
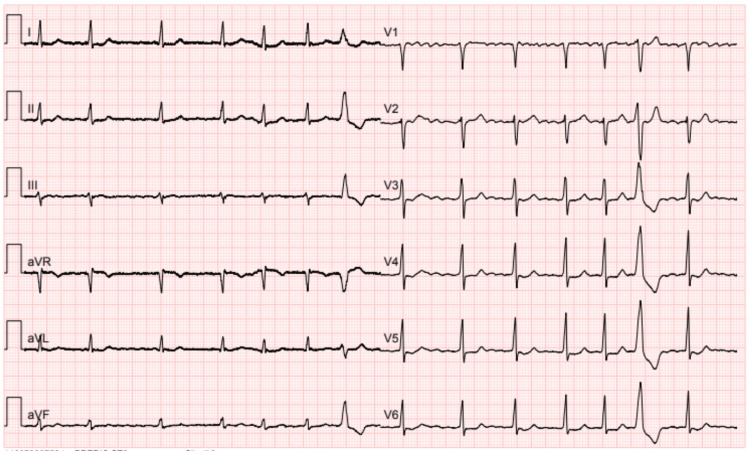
A 12-lead electrocardiogram upon admission A 12-lead electrocardiogram showed atrial fibrillation with normal intraventricular conduction and one single premature ventricular complex.

A 74-year-old male presented to the emergency department with palpitations lasting less than 48 hours. An electrocardiogram revealed atrial fibrillation, as shown in Figure 1.

Because of his age, hypertension, diabetes, and peripheral arterial disease, his congestive heart failure, hypertension, age ≥75, diabetes, stroke, vascular disease, age between 65-74, and female sex (CHA2DS2-VASC) score was 4. The patient also suffered from von Willebrand disease (vWD), diagnosed at the age of 58. Since then, the patient has received treatment with factor VIII (FVIII)/vWF supplementation; however, severe bleeding episodes have recurred despite treatment. He had no family history of bleeding disorders and no past medical history of severe bleeding episodes before the age of 58. Anticoagulation was withheld, and a pharmacological strategy for rhythm control was pursued. LAAC was considered for stroke prevention. A detailed assessment of his coagulation cascade was performed before the procedure. FVIII was 10%, vWF antigen was 16%, and vWF activity was <3% (FVIII, vWF antigen, and vWF activity normal values are 50%-150%). Due to the decreased levels of FVIII and vWF activity, the procedure was delayed. Because of persistently low levels of FVIII levels and vWF activity despite adequate FVIII supplementation, an acquired form of vWD was suspected. Protein electrophoresis revealed a monoclonal gammopathy of undetermined significance with IgG prevalence. To test whether the MGUS could have caused vWD, a full dose of FVIII and vWF was administered, and FVIII%, vWF antigen, and vWF activity were measured at one and four hours. After one hour, FVIII was 35%, vWF antigen was 106%, and vWF activity was 32%. After four hours, FVIII was 20%, vWF antigen was 52%, and vWF activity was 9%. Due to the rapid decrease in vWF activity and FVIII% immediately after FVIII/vWF administration, AvWS was suspected, and intravenous immunoglobulins (IVIG) were started. Good levels of FVIII%, vWF antigen, and vWF activity were acquired after IVIG administrations. Rapid clearance of factor VIII and vWF, in addition to good responses to IVIG, were compatible with a diagnosis of AvWS secondary to MGUS.

**Figure 2:**
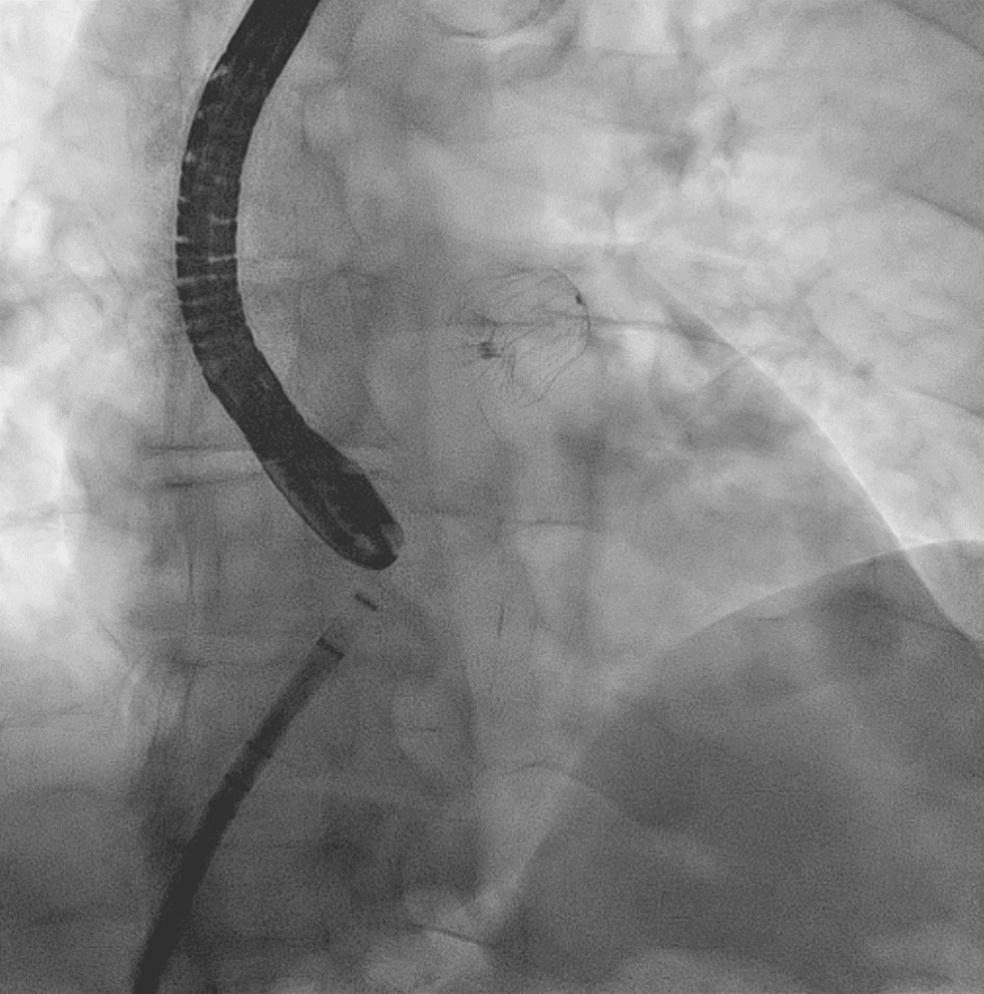
The deployed left atrial appendage occluder Echocardiographic monitoring confirmed the correct positioning of the device.

Thereafter, LAAC was performed with the implantation of a WATCHMAN FLX™ 24mm (Boston Scientific Corporation, Marlborough, Massachusetts, United States). There were no intra- or post-procedural complications. Correct positioning was confirmed by transesophageal echocardiography, as shown in Figure 2.

The patient was discharged on low-dose aspirin daily for three months and IVIG every 15 days. Aspirin was stopped after three months. At nine-month follow-up, the patient was well and did not report embolic or hemorrhagic episodes.

## Discussion

**Figure 3:**
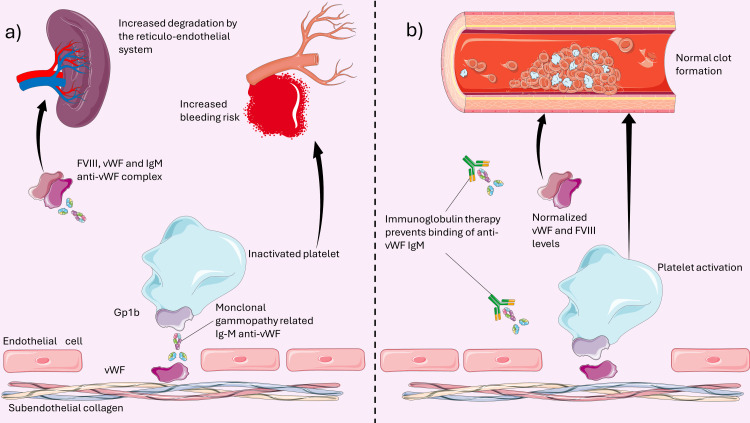
Pathophysiology of acquired von Willebrand disease and therapeutic action of immunoglobulins Figure a illustrates the pathophysiology of acquired von Willebrand disease. Monoclonal gammopathy-related anti-von Willebrand factor IgM binds to von Willebrand factor (vWF) both at the level of subendothelial collagen and when bound to factor VIII (FVIII), causing an increased degradation of the FVIII-vWF complex. The inability of vWF to interact with Gp1b does not allow platelet activation and therefore increases bleeding risk. Figure b shows the mechanism through which immunoglobulins provide a therapeutic benefit. Binding to the anti-vWF IgM allows normal platelet activation and prevents the degradation of the FVIII-vWF complex. Image credit: Alessandro Giaj Levra. The figure was drawn using Server Medical Art. Servier Medical Art by Servier is licensed under a Creative Commons Attribution 3.0 Unported License.

The present report exemplifies stroke prevention with percutaneous LAAC in a patient with high bleeding risk secondary to AvWS. Furthermore, it suggests an approach to the pre-procedural management of patients with AvWS. There is scant data regarding the optimal management of AF in patients with AvWS. Despite their increased bleeding risk, these patients can still carry a high thromboembolic risk and should receive stroke prevention measures [7]. Currently, administering OACs is suggested over no treatment in patients with vWD [8]. However, this may further increase their hemorrhagic risk and must be carefully evaluated [9,10]. IVIG has shown promising results for reducing the bleeding risk in patients with AvWS secondary to MGUS. The mechanism of action of IVIG in AvWS is shown in Figure 3.

Alternatively, 1-deamino-8-D-arginine vasopressin (DDVAP) has shown to be effective in controlling minor bleeds [11]. The main pitfall of current therapies are the temporary nature of their effects. Therefore, patients being treated for AvWS secondary to MGUS with concomitant AF have a continuous shift between an increased hemorrhagic and thrombotic risk, thus potentially rendering OAC both beneficial and harmful.

LAAC in patients with AvWS and AF may be a safe option to balance the hemorrhagic and thrombotic risks.

For stroke prevention in AF, alternative treatments such as catheter ablation typically necessitate the continuation of OAC therapy for two weeks post-procedure. The continuation of OAC therapy is subsequently guided by the CHA2DS2-VASC score. Given the increased thromboembolic risk, the patient was assessed to require life-long anticoagulation. Consequently, considering the patient's initial AF episode, elevated bleeding risk, patient preference, and the necessity for OAC therapy, catheter ablation was not performed.

To the best of our knowledge, this is the first reported case of LAAC in a patient with AvWS secondary to the MGUS IgG subtype and AF. LAAC has been performed in other bleeding disorders such as hemophilia, congenital vWD, and myelodysplastic syndromes. Dognin et al. reported good results in terms of safety and effectiveness of percutaneous LAAC closure in primary hemostasis disorders in a single-center registry. Patients with primary vWD received preoperative desmopressin or no treatment; a WATCHMAN^TM^ device was implanted, and aspirin was used as a single antiplatelet therapy at discharge [11]. 

Güray et al. reported successful LAAC in a patient with hemophilia A [12]. Preprocedural factor VIII was administered, an AMPLATZER AMULET^TM^ device was used, and one-month double antiplatelet therapy with clopidogrel and low-dose aspirin was the antithrombotic therapy of choice.

Bhatti et al. also reported a case of percutaneous LAAC in a patient with hemophilia B [13]. Preoperative management included warfarin and recombinant factor IX; a WATCHMAN^TM^ device was used; and antithrombotic therapy was discontinued after one month. However, the patient experienced a transient ischemic attack, demonstrating an increased thromboembolic risk.

The aforementioned cases demonstrate the safety of the procedure in patients with hemostasis disorders, with gastrointestinal bleeding secondary to transesophageal echocardiography being the only complication reported [11]. Great variability exists in terms of preoperative treatment, choice of the device used for LAAC, and post-operative antithrombotic therapy, with no evidence available to date to guide the management of these patients.

## Conclusions

Although AvWS secondary to MGUS is a rare condition, management of AF in these patients is challenging. Patients with AvWS and AF carry both an increased bleeding and thromboembolic risk. LAAC may be a safe alternative to OAC for patients who cannot maintain life-long anticoagulation.

The use of pre-procedural immunoglobulins decreases the hemorrhagic risk of patients with AvWS. Routine pre-treatment with IVIG may improve surgical outcomes in this population. However, larger studies are required to assess the efficacy of this strategy.
